# PEGylation and folic-acid functionalization of cationic lipoplexes—Improved nucleic acid transfer into cancer cells

**DOI:** 10.3389/fbioe.2022.1066887

**Published:** 2022-12-21

**Authors:** Marco Hoffmann, Sven Gerlach, Christina Hoffmann, Nathalie Richter, Nils Hersch, Agnes Csiszár, Rudolf Merkel, Bernd Hoffmann

**Affiliations:** Institute of Biological Information Processing, Mechanobiology (IBI-2), Research Center Juelich, Juelich, Germany

**Keywords:** DNA-transfer, PEGylation, selective nucleic acid transfer, biocompatibility, functionalized lipoplexes

## Abstract

Efficient and reliable transfer of nucleic acids for therapy applications is a major challenge. Stabilization of lipo- and polyplexes has already been successfully achieved by PEGylation. This modification reduces the interaction with serum proteins and thus prevents the lipoplexes from being cleared by the reticuloendothelial system. Problematically, this stabilization of lipoplexes simultaneously leads to reduced transfer efficiencies compared to non-PEGylated complexes. However, this reduction in transfer efficiency can be used to advantage since additional modification of PEGylated lipoplexes with functional groups enables improved selective transfer into target cells. Cancer cells overexpress folate receptors because of a significantly increased need of folate due to high cell proliferation rates. Thus, additional folate functionalization of PEGylated lipoplexes improves uptake into cancer cells. We demonstrate herein that NHS coupling chemistries can be used to modify two commercially available transfection reagents (Fuse-It-DNA and Lipofectamine^®^ 3000) with NHS-PEG-folate for increased uptake of nucleic acids into cancer cells. Lipoplex characterization and functional analysis in cultures of cancer- and healthy cells clearly demonstrate that functionalization of PEGylated lipoplexes offers a promising method to generate efficient, stable and selective nucleic acid transfer systems.

## Introduction

The development of stable and efficient delivery systems for nucleic acids is of critical importance for biotechnological and biomedical applications (Gencer et al., 2020; Mohanty et al., 2020)*.* Local *in vivo* applications additionally often depend on small volumes (3–10 µl) with high nucleic acid concentrations (Walther et al., 2004; Geetha-Loganathan et al., 2011). Underlying protocols for applications in mammals and human therefore differ from those for classical cell culture transfection systems as also shown by us for local treatment of glioblastoma in mice (Hoffmann et al., 2022). Beside their intense utilization in anti-cancer treatments (Amreddy et al., 2018), nucleic acid therapies can also be successfully used for immunization, as demonstrated in the current Covid pandemic *via* a SARS-CoV-2 mRNA vaccine (Chung et al., 2020; Polack et al., 2020). One of the most crucial developments for lipoplex formulations in organisms is their stabilization by polyethylene glycol (PEGylation) (Ogris et al., 1999; Yu et al., 2004)*.* Here, complexes made of nucleic acids and lipid formulations or polymers are additionally modified to form a protective PEG shell around these systems (Wang et al., 2012)*.* Modification causes reduced recognition and protein adsorption *in vivo* by avoiding the Mononuclear phagocyte system (MPS), also known as reticuloendothelial system (RES) responsible for nanoparticle elimination from circulation (Li and Huang, 2009; Guo and Huang, 2011; Gao et al., 2014)*.* Thereby the half-life of PEGylated lipoplexes can be extended up to 30-fold (Anselmo et al., 2015). The first PEGylated liposomal formulation, Doxil^®^ was approved by the Food and Drug Administration (FDA) already in 1995. Doxil “Stealth^®^” lipoplexes displayed a drug half-life of 72 h and circulation half-life of 36 h (Laginha et al., 2005; Salvatorelli et al., 2012). PEGylation can be combined with further tunctionalization, as e.g. pH sensitive coupling such as hydrazone, to enhance selective release of therapeutics into tumor tissue (Chan et al., 2012).

However, PEGylation results in a significant reduction of lipoplex transfer efficiency also known as PEG-dilemma (Mishra et al., 2004; Hatakeyama et al., 2013; Ge et al., 2014; Fang et al., 2017). Different strategies attempted to circumvent this reduction, either by use of pH sensitive and cleavable PEG molecules (Fang et al., 2017) or by variations in PEG chain-length and concentration (Pozzi et al., 2014; Nosova et al., 2019; Khan et al., 2020). Variations in PEG concentration additionally influence the conformation of the resulting PEG shell (Yang et al., 2014). Here, with low concentrations used (less than 5 mM), neighboring PEG chains do not overlap to form a separated, so-called “mushroom” conformational regime. At increased PEG concentrations above 5 mM, PEG arms can no longer interact with the liposome surface, resulting in a so-called “brush” shape (DeRouchey et al., 2006), with PEG arms protruding from the liposome surface. At even higher PEG-concentrations, free PEG micelles are formed (Nosova et al., 2019).

PEGylation additionally leads to reduced zeta potentials of lipoplexes (Pozzi et al., 2014). This reduction generally deteriorates uptake due to impaired interaction with the cell membrane (Resina et al., 2009). In contrast, the particle size remains almost unchanged, thus maintaining a crucial property of lipoplexes for efficient transfers of nucleic acids (Ren et al., 2019; Hoffmann et al., 2020). For such PEGylation, a certain formulation of lipoplexes can be modified by N-Hydroxysuccinimide (NHS)-chemistry, depending on their specific molecular composition (Yu et al., 2004; Ikeda and Nagasaki, 2014). This chemistry covalently binds NHS-coupled functional groups, such as PEG (NHS-PEG) to primary amines (-NH_2_), present in many lipids (e.g. DOPE) used in lipoplex formulations (Ma et al., 2007; Csiszar et al., 2010; Cardarelli et al., 2016; Kolasinac et al., 2018; Hoffmann et al., 2020). As a result, stable functionalization with pre-existing lipoplexes can be produced by the so-called post-insertion method (Nosova et al., 2019)*.*

In the last decades, the use of gene therapies against cancer increased significantly. Due to their high metabolic activity, cancer cells often overexpress various surface proteins, as e.g. HER2, transferrin or folate receptors (Cheung et al., 2016; Pieroth et al., 2018; Tian et al., 2022). Various therapeutic delivery approaches have already been attempted to use especially the folate receptor for targeted delivery (Ledermann et al., 2015; Pal et al., 2017; Son et al., 2018). Therefore, the previously described PEGylation combined with folate functionalization could allow targeted transfer of nucleic acids (e.g., pDNA) into cancer cells. Besides breast cancer, glioblastoma belongs to the most threatening and deadliest cancers worldwide (Davis, 2016; Taylor et al., 2019). A promising treatment method is not yet available, so that local dissections in combination with irradiation and chemotherapy are currently applied as first line treatment to result in an averagedprolongation in life of 12 months (Zhang et al., 2019)*.* Breast cancer is the most frequent cause of cancer death in women. The effective treatment in most cases consists of irradiation, chemotherapy and amputation (Lu et al., 2009). The development of effective and at the same time stable nucleic acid delivery systems offers a promising new route for treatment of such diseases. For optimal development and adaptation of such delivery systems, analyses should ideally be based on comparable *in vitro* systems (Shibata et al., 2015; Silverman and Barenholz, 2015). Here we have developed functionalized lipoplexes based on commercially available transfer systems. We use one of the most widely used systems (Lipofectamine^®^ 3000), which transfers nucleic acids *via* endosomal uptake into cells. In comparison, we functionalize a novel transfer system (Fuse-It-DNA) that transfers nucleic acids directly into the cytoplasm *via* fusion of lipoplexes with cellular plasma membranes (Hoffmann et al., 2019; Hoffmann et al., 2020). We functionalize both systems by NHS-coupled PEGylation and characterize resulting effects on chemical and functional level with special focus on cancer cell targeting. Furthermore, we put special emphasis on a simple transferability of the overall system from *in vitro* cell culture analyses to later *in vivo* applications.

## Material and methods

### Cell culture

Two different cancer cell types were used. For a glioblastoma *in vitro* cancer model, the cell line U87 (ATCC^®^, HTB-14™, derived from human) was compared with freshly isolated cortical neurons from E18 old rat embryos. F98 glioblastoma cells (ATCC^®^, CRL-2397™, derived from rat) were used as folate receptor positive cancer cells for western blot analysis to characterize antibody cross reactivity for human and rat cells (Supplementary Figure S1). For a breast cancer *in vitro* model, the breast cancer associated cell line MCF-7 (ATCC^®^, HTB-22™, derived from human) was utilized in comparison to primary human foreskin fibroblast (HFF) (ATCC^®^, SCRC-1041™, derived from human).

All cell types were cultivated as indicated in Table 1 at 37°C in a humidified atmosphere containing 5% and CO_2_. Cultivation of U87, MCF-7, HFF and F98 cells were performed by exchanging cell culture medium every 2 to 3 days. At a cell density of 80%–90%, cells were subcultured by incubation with 0.05% trypsin-EDTA solution (Sigma Aldrich, United States) at 37°C for 5–7 min. Isolation and cultivation of primary cortical neurons was performed as described previously (Fricke et al., 2011) and 1.2 × 10^6^ neurons were seeded on 0.01% (v/v) Poly-l-Lysin (PLL) (Sigma Aldrich, United States) coated 24-well plates (Thermo Scientific, United States). Neuronal network formation was performed under medium change every 2–3 days for 11 days until transfection. U87 (70.000 cells/cm^2^), MCF-7 (80.000 cells/cm^2^) and HFF (40.000 cells/cm^2^) cells were seeded on 0.01% (v/v) human fibronectin (FN) (VWR, United States) coated 24-well plates 24 h before transfection. At time of transfection, all cells were 70%–90% confluent. For western blot analyses all cell types as well as F98 cells (90.000 cells/cm^2^) were seeded as described above.

**TABLE 1:** Cell culture medium for assessed cell types. All media and supplements were purchased from either Thermo Fischer^a^ (United States) or Sigma Aldrich (United States)^b^.

*In vitro* cancer model	Cell type	Media	Supplemented with
Glioblastoma	U87	Minimum Essential Medium^b^	10% of fetal bovine serum^a^ 1% of 10.000 U Penicillin-Streptomycin^a^ 1% of 100x non-essential amino acids^b^ 1% of 100x L-glutamine solution^a^
	pr. cortical neurons	Neurobasal Medium^a^	2% of 100x B-27 supplement^a^ 0.25% of 200 mM GlutaMax^a^ 0.1% of 50 mg/ml gentamicin reagent solution^(a)^
	F98	Dulbecco Modified Eagle Medium GlutaMax^a^	10% of fetal bovine serum^a^ 1% of 10.000 U Penicillin-Streptomycin^a^
Breast cancer	MCF-7	RPMI Media 1,640 GlutaMax^a^	10% of fetal bovine serum^a^ 1% of 10.000 U Penicillin-Streptomycin^a^ 1% of 100x non-essential amino acids^b^ 1% of 100 mM sodium pyruvat solution^a^ 0.5% of insulin human^b^
	HFF	Dulbecco Modified Eagle Medium GlutaMax^a^	10% of fetal bovine serum^a^ 1% of 10.000 U Penicillin-Streptomycin^a^

The amounts indicated are given as (v/v).

#### Preparation of Fuse-It-DNA and Lipofectamine^®^ 3000

In this study two commercially available cationic transfection reagents, namely Fuse-It-DNA (Beniag GmbH, Germany) and Lipofectamine^®^ 3000 (Thermo Scientific, United States) were functionalized. Preparation of fusogenic lipoplexes (Fuse-It-DNA) and endocytic lipoplexes (Lipofectamine^®^ 3000) were performed according to the manufacturer’s instruction for transfer of 1 µg plasmid DNA (pDNA) and analyzed on both *in vitro* cancer models (here described as *in vitro* protocol). To allow a prospective *in vivo* application, both systems have additionally been optimized to reduced volumes with high transfer efficiencies and biocompatibilities (here described as *in vivo* protocol).

For Fuse-It-DNA *in vivo* protocols OptiMEM dilution was omitted. Therefore, analog to the *in vitro* protocol, 1 µg enhanced green fluorescent protein pDNA (eGFP-pDNA) or constitutively active Caspase3 pDNA (ca-Caspase3-pDNA) was complexed with 2 µl of Fuse-It-DNA containing neutralization buffer (NB) at room temperature for 10 min. After incubation, 2.5 µl of fusogenic solution (FS) was added and mixed by vortexing for 10 s. This solution (5.5 µl) was directly transferred into 1 ml cell culture medium for 24 h. For lipofection *in vivo* protocols, 2.5 µg eGFP-pDNA or ca-Caspase3-pDNA were incubated with 2.5 µl of P3000^®^ reagent at room temperature for 10 min. Then 5 µl of Lipofectamine^®^ 3000 reagent was added, and the solution was mixed by vortexing for 30 s. Subsequently, another incubation step was performed at room temperature for 30 min. For transfer of 1 µg plasmid DNA, 4 µl of this solution was pipetted into 500 μl cell culture medium.

#### Preparation of PEG and PEG-FA conjugated Fuse-It-DNA and Lipofectamine^®^ 3000

For the modification and functionalization of *in vivo* protocols, Fuse-It-DNA and Lipofectamine^®^ 3000 were PEGylated by using the post-insertion method (Nosova et al., 2019)*.* According to this method, endocytic and fusogenic lipoplexes were either loaded with eGFP-pDNA or ca-Caspase3-pDNA as described above. Then, the outer lipid bilayer of the pDNA-loaded lipoplexes was modified by adding 0.4–12 mM, in a total volume of 1 μl HO-PEG5K-NHS (PEG) (Sigma Aldrich, United States) or Folate-PEG5K-NHS (PEG-FA) (NanoSoft Polymers, United States) to reach final concentrations of PEG/PEG-FA from 0.1–3 mM for lipofection and 0.06–2.3 mM for fusion. pDNA-loaded lipoplexes were incubated with PEG/PEG-FA at room temperature for 5 min to ensure optimal functionalization. Subsequently, transfection volumes of both systems were adjusted to transfer 1 μg pDNA for each sample.

#### Reduction of FolR1-α surface binding capacity

FolR1-α dependent transfer of folate modified eGFP-pDNA-containing lipoplexes was evaluated by reducing the lipoplex binding probability to FolR1-α in the presence of uncoupled folic acid (FA) (Sigma Aldrich, United States). For this purpose, 1 mM FA was added to the corresponding cell culture medium of both *in vitro* cancer models. Cells were pre-incubated at 37°C for 2 h until transfection. Preparation of *in vivo* protocols for both systems was performed as described above. Throughout the whole transfection time for 24 h 1 mM FA was present.

#### Confocal light microscopy

Live cell analysis and visualization of the fluorescent eGFP signal was performed 24 h after treatment with Fuse-It-DNA and Lipofectamine^®^ 3000 by using an inverse confocal laser scanning microscope (cLSM 710, Carl Zeiss Jena, Germany). All overview images were recorded in the center of the substrate with an EC “Plan-Neofluar” 10x/0.30 Ph1 air objective (Carl Zeiss Jena, Germany). For all experiments eGFP fluorescence was excited by a 488-nm argon ion laser and detected through a variable transmittance band pass filter adjusted to 500–550 nm. All microscope settings were kept identically to ensure comparability throughout all experiments.

### Analysis of DNA transfer efficiencies by flow cytometry

Immediately after live cell analysis, the flow cytometer (CytoFLEX S Flow Cytometer, Beckmann Coulter) was used to determine eGFP-pDNA transfer efficiencies for all transfections. For flow cytometric analysis, all cells were trypsinized with 250 μl 0.05% trypsin-EDTA solution (Sigma Aldrich, United States) at 37°C for 3 min and centrifuged with 300 g. To allow cell separation of neuronal networks, cells were centrifuged with 200 g for 5 min. Cell populations were gated separately based on cell granularity and size (forward (FSC) and side scatter (SSC)). At least 10.000 cells were analyzed by using appropriate filter settings to determine eGFP transfer efficiencies.

### Characterization of ca-Caspase3 functionality *via* a flow cytometric based live/dead assay

Evaluating the cell viability by flow cytometry 24 hpt the “LIVE/DEAD™ Fixable Violet stain fluorescence assay” (L34963, Invitrogen, United States) was used. In principal living cells react with the kit’s fluorescent reactive dye on their surface resulting in a weak fluorescent signal while dead cells with compromised plasma membrane incorporate the dye within the cytosol showing a bright signal. To measure all dead cells the supernatant of the substrate was involved and centrifuged before trypsinization. For precise differentiation between live and dead cells, a positive dead control (treated with 1 ml ethanol p.a. for 20 min at RT) was used. However, all cell viability stainings were performed according to the suppliers’ given protocols. During flow cytometry the cell populations were then gated separately based on cell granularity and size FSC and SSC . At least 10.000 cells were analyzed by using appropriate laser and filter settings to determine ca-Caspase3 activity.

### Complex characterization by size- and zeta potential distribution measurements and *in situ* long term stability

Size- and zeta potential distribution were determined by dynamic and electrophoretic light scattering, respectively using a ZetaSizer (ZetaSizer Nano ZS from Malvern Instruments, Malvern, United Kingdom) equipped with a HeNe laser (633 nm). Scattered laser light was collected at a constant angle of 173°. Measurements were performed with both transfection reagents and their functionalized complexes for *in situ* long term stability. The preparation of lipoplexes for characterization of both transfection reagents and their functionalized complexes were performed as described previously. For *in situ* complex analyses, only the *in vivo* protocol was used with and without PEG-FA functionalization and prepared as shown before. The complexes were stored at 4°C after preparation and measured in an interval of 3 days for 4 weeks. For all characterization, the solutions were diluted 1/10 for size distribution and 1/100 for zeta potential measurements with purified, degassed, and sterile filtered water (Milli-Q Gradient A10, Merck Millipore, Germany). Size was determined by intensity and zeta potential measurements were performed with a refractive index of 1.33. All measurements were performed at 21°C and repeated three times at 1 min interval. Data were collected from three independently prepared samples and analyzed using the instrument software (DTS from Malvern Instruments).

### Protein isolation and FolR1-α quantification *via* western blot

Quantification of FolR1-α protein levels was performed *via* western blot analysis for all cell types. Therefore, cells were cultured in 12-well plates (Thermo Scientific, United States) to 80%–90% confluency. After washing with cold 1x PBS, pH 7.4 (ThermoFischer, United States), 250 μl of RIPA lysis-buffer (Sigma Aldrich, United States) supplemented with 1% protease inhibitor (v/v) (Sigma Aldrich, United States) was added to each well. Cell lysis was performed at room temperature for 20 min. Subsequently, the lysates were centrifuged at 16.000 g for 10 min at 4°C and 200 μL of each supernatant were heated to 95°C for 5 min after addition of 50 μl 5x loading dye (Bio-Rad, United States). 15 μL of each protein isolate was fractionated at 100 V for 2 h in ready-to-use 4%–20% gradient gels (Mini-Protean, Bio-Rad, United States) by SDS-PAGE and blotted to a nylon membrane. Using Anti-Tubulin Y1/2 clone (MAB 1864, Millipore, United States) as constitutively expressed marker, overall protein level of FolR1-α was determined with an anti-Folate Binding Protein/FBP antibody (ab221542, Abcam, United States). Secondary antibody Anti-Rat (A8438, Sigma Aldrich, United States) and Anti-Rabbit (A3812, Sigma Aldrich, United States) coupled to alkaline phosphatase were visualized by incubation of NBT/BCIP substrate solution (Thermo Scientific, United States) at room temperature for 5 min.

### Statistical and graphical evaluation

All data are given as mean including standard deviation of at least three independent measurements. Relative calculated data were generated from each experiment and combined accordingly from the different individual experiments to give a relative mean and the corresponding standard deviation. Statistical analysis (univariate ANOVA) was performed with Microsoft Excel (MS Office 2019) for multiple comparisons. Figures and graphs were created using Origin 2019 64 Bit (Origin Lab Graphing & Analysis). Only significant differences between individual probes were marked in graphs with asterisks. For this purpose, a *p*-value of ≤.05 was considered as significant. *p*-values of 0.05, 0.01 and 0.001 were labeled with one to three asterisks.

## Results

### Utilization of FR overexpression in cancer cells for targeted deliveries

**FIGURE 1:**
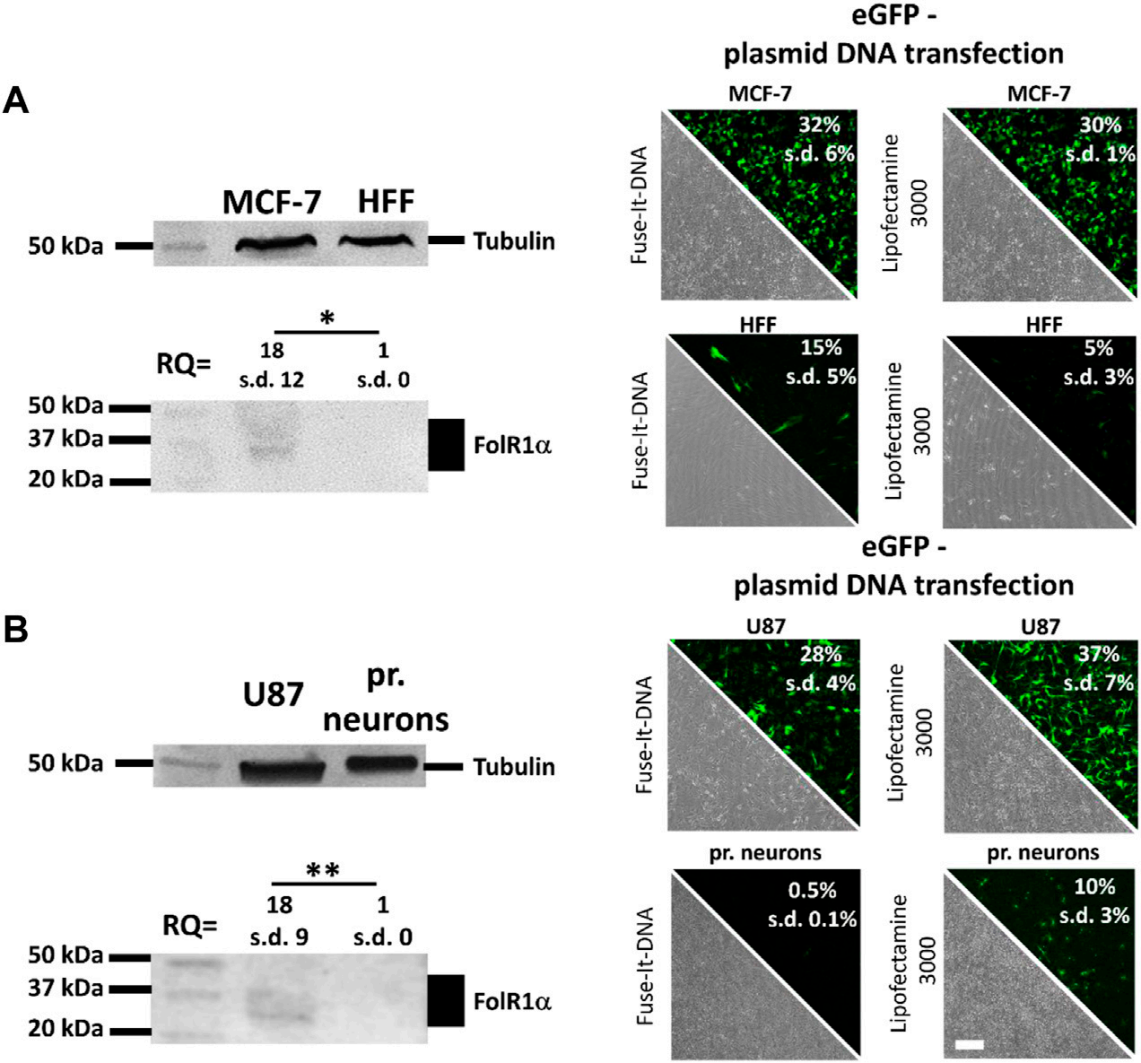
Quantification of folate receptor one alpha (FolR1α) protein levels and DNA transfer into healthy and cancer cells. FolR1α was quantified by western blot in MCF-7 breast cancer cells and healthy primary human fibroblasts (HFF) **(A)** as well as U87 glioblastoma cells and healthy primary cortical neurons **(B)**. The relative quantities (RQ) were adjusted to tubulin and the resulting ratios were analyzed for significance. For all cell types, transfection efficiency was determined using Fuse-It-DNA and Lipofectamine^®^ 3000 with transfer of an eGFP encoding plasmid. Results were recorded by fluorescence microscopy (phase contrast and eGFP channel) and flow cytometry. Scale bar 200 μm. *n* = at least three independent experiments.

Cancer cell targeting depends on specific molecules present only in those cells. Since cancer cells are known to overexpress folate receptors (FR), we tested two different cancer cell lines for FR protein levels. Western blot analysis showed for breast cancer MCF-7 cells as well as for the glioblastoma U87 cells a clearly detectable FR signal. In contrast no signal was detectable in non-cancerous HFF cells and primary neurons which were chosen as controls (Figure 1A, B). For simplicity reasons only, cancer cells together with chosen controls were subsequently named as “breast cancer *in vitro* model” and “glioblastoma *in vitro* model”, respectively. Antibody cross-specificity between cells of different species was demonstrated and shown in Supplementary Figure S1. Since cancer cells (MCF-7 and U87) show a distinct and significant 18-fold increase in FR expressions compared to healthy primary cells (HFF and primary neurons) (Figure 1A), FA-functionalization of nucleic acid transfer systems could therefore lead to an improvement of transfection efficiencies that is supposed to be reduced by pure PEGylation in healthy and cancer cells.

Using fusogenic (Fuse-It-DNA) and endocytic lipoplexes (Lipofectamine^®^ 3000) (Figure 1), transfer efficiency of both reagents was analyzed in all cell types. Using Fuse-It-DNA, markedly enhanced transfer efficiencies for MCF-7 cancer cells and more biocompatible transfer in respective HFF control cells was demonstrated compared to lipofectamine^®^ 3000. For the glioblastoma model contrary results were found. Here, Lipofectamine^®^ 3000 showed higher efficiencies for U87 cancer cells and primary neurons. Both transfer systems were therefore used for further functionalization and characterized for targeting abilities in the respective *in vitro* model. The morphology of all cell types after transfection with Fuse-It-DNA (MCF7/HFF) and Lipofectamine^®^ 3000 (U87/pr. neurons) is shown in high resolution in Supplementary Figure S2.

### Development of efficient, *in vivo* applicable protocols - Effects of PEG-FA modifications on Fuse-It-DNA and Lipofectamine^®^ 3000

**FIGURE 2:**
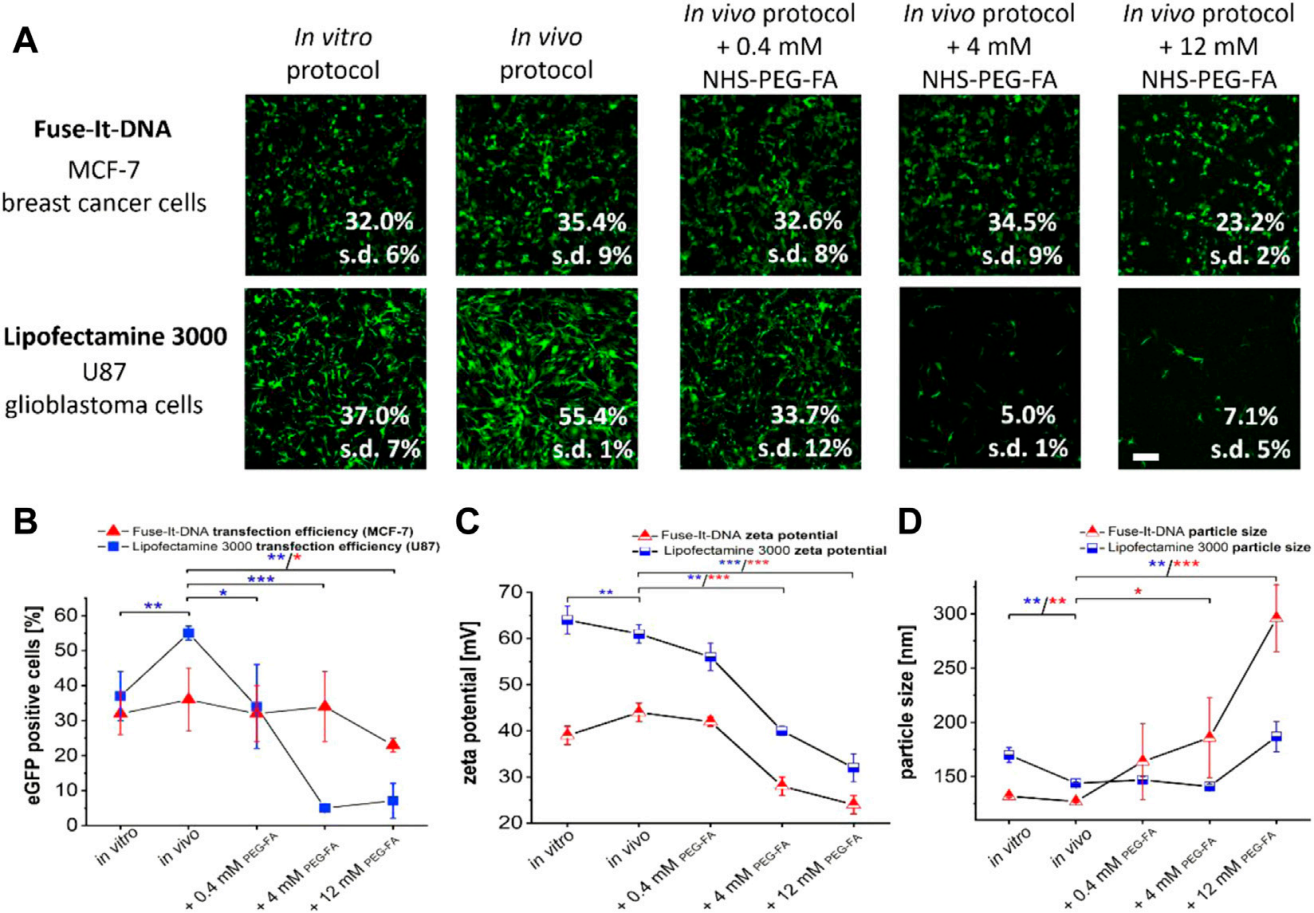
Development of *in vivo* applicable transfection protocols and functionalization of Fuse-It-DNA as well as Lipofectamine^®^ 3000 with PEG-FA. To optimize manufacturer’s transfection protocols (*in vitro* protocols) for *in vivo* applications, protocols were established that require minimum volumes to transfer 1 μg of plasmid (*in vivo* protocol). In addition, lipoplexes were functionalized with different molarities of NHS-PEG-FA-stocks with concentrations between 0.4 to 12 mM by post modification. Analysis of transfections was performed by fluorescence microscopy and flow cytometry **(A)**. The influence of PEG-FA modification on transfer efficiencies is shown in **(B)**. Unmodified complexes as well as the PEG-FA modified complexes were characterized for zeta potential **(C)** and complex size **(D)**. Scale bar 200 μm. *n* = at least three independent experiments.

Local *in vivo* delivery often depends on small transfer volumes that cannot be reached with standard transfection protocols. This is not only important for breast cancer, but even more essential for glioblastoma treatment (Hoffmann et al., 2022). We therefore additionally adapted standard cell culture *in vitro* protocols (Figure 2A, “*in vitro*”) with high transfection volumes of 50 µl per 1 µg of DNA in such a way that significantly reduced volumes of 4 µl (Lipofectamine^®^ 3000) and 5.5 μl (Fuse-It-DNA) could be applied for possible local applications *in vivo*. Although also for the adapted protocols all experiments very performed in cell culture, we termed it in the following “*in vivo”* for better differentiation (Figure 2A). As a result, both adapted *in vivo* protocols showed improved transfer efficiencies and significantly higher numbers of vital cells compared to standard protocols (*in vitro*) 24 h after DNA transfer.

Additional functionalization of lipoplexes of both systems with PEG-FA in concentrations between 0.4 mM and 12 mM showed different effects for the two transfection reagents. For Fuse-It-DNA, largely unaffected transfer efficiencies were observed at low and intermediate PEG-FA concentrations. Only functionalization with high 12 mM PEG-FA concentrations showed significant reductions in transfer efficiencies (Figure 2A, B). In contrast, for Lipofectamine^®^ 3000 already low concentrations of 0.4 mM PEG-FA resulted in a significant reduction in transfer efficiency. The effect was strongly enhanced at higher concentrations with up to 90% in U87 glioblastoma cells.

For lipoplex characterization and analysis of the zeta potential, both systems showed comparable reductions in zeta potentials upon functionalization with increasing PEG-FA concentrations (Figure 2C). Main differences between both systems were mainly an overall lower zeta potential by 15–20 mV for all Fuse-It-DNA modifications compared to the respective lipofectamine lipoplexes. The complex size of Fuse-It-DNA showed a steadily increasing trend in size distributions with increasing PEG-FA concentration, doubling in size from approximately 130 nm to complexes above 300 nm for 12 mM PEG-FA. In contrast, Lipofectamine^®^ 3000 complexes did not change significantly with increasing PEG-FA up to concentrations of 4 mM, whereas the size of resulting complexes with 12 mM PEG-FA significantly increased.

### Improvement of transfer efficiencies in cancer cells by PEG-FA conjugated lipoplexes compared to pure PEGylation

**FIGURE 3:**
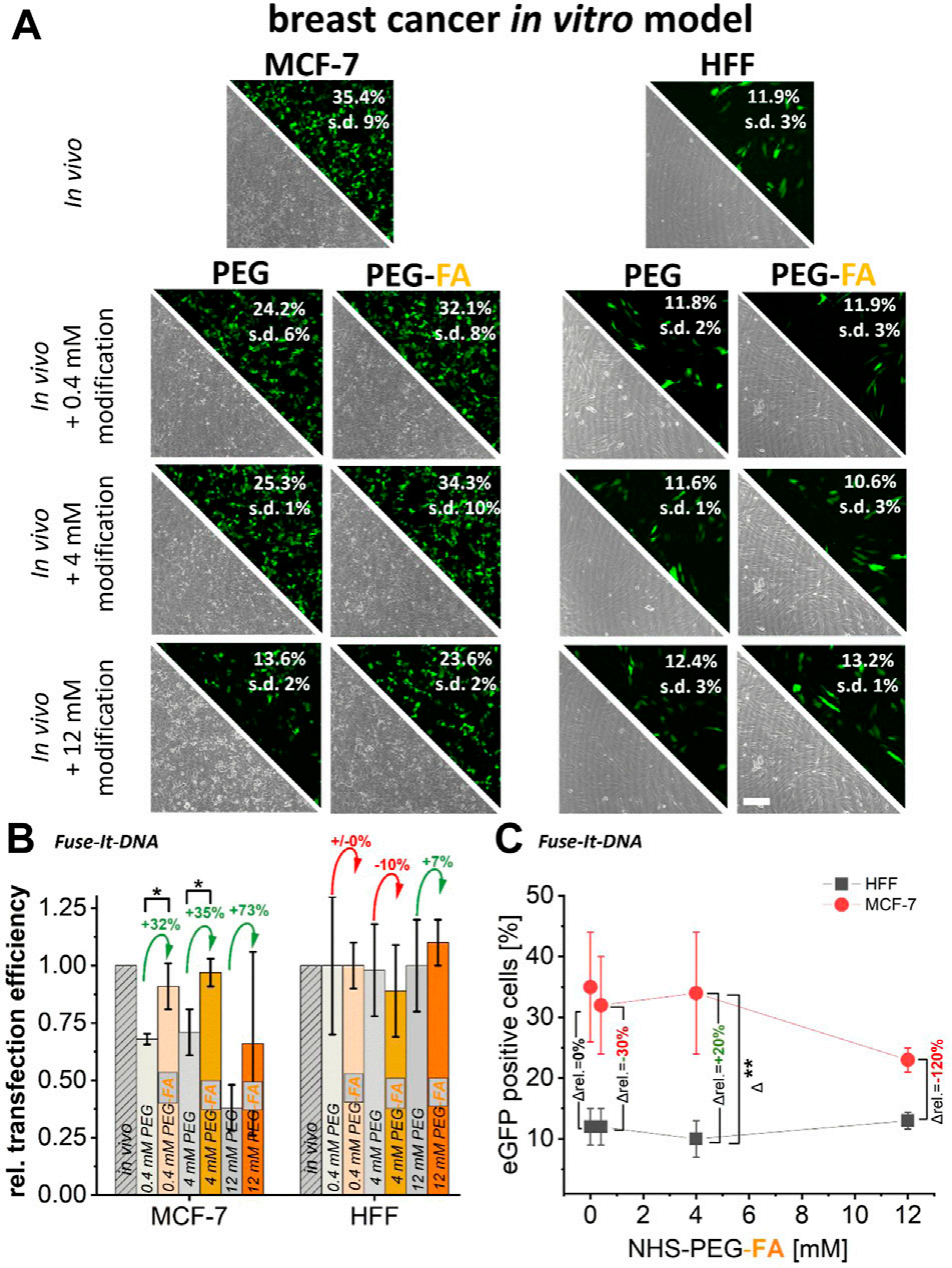
Effect of pure PEGylation and additional FA-functionalization on transfer efficiencies. MCF-7 and HFF cells, were transfected with eGFP plasmid using Fuse-It DNA (*in vivo* protocol). In addition, this lipoplexes were further functionalized with PEG and PEG-FA at equal concentrations. Analysis was performed by fluorescence microscopy and flow cytometry **(A)**. To demonstrate the supplemental effects of FA, relative transfection efficiencies are plotted in **(B)** and alterations of mean values are shown in green (positive) and red (negative). In **(C)**, progressions of transfection efficiencies by PEG-FA with improvements in favor of cancer cells (green) and deteriorations (red) are shown. Scale bar 200 μm. *n* = at least three independent experiments.

To analyze the specificity of PEG-FA modifications and the resulting cancer cell targeting abilities, PEG-modifications for both *in vitro* cancer models were used with and without the functional FA-group for NHS coupled-modifications. For Fuse-It-DNA, significantly higher transfer efficiencies by 30%–70% were achieved in MCF-7 cancer cells with 0.4 mM and 4 mM of PEG-FA functionalized complexes compared to pure PEGylation. Relative efficiencies increased continuously with increasing concentration of FA functionalization (Figure 3A, B). Moreover, up to a concentration of 4 mM PEG-FA, transfer efficiencies were comparable to those obtained in the unmodified *in vivo* protocol. In contrast, for healthy HFF cells, no significant improvements in transfer efficiency could be detected for FA-functionalized lipoplexes compared to pure PEGylation. Comparing transfer efficiencies with increasing PEG-FA modifications between healthy HFF cells and MCF-7 cancer cells (Figure 3C) showed significantly enhanced transfer efficiency in favor of MCF-7 cancer cells with 4 mM PEG-FA by 20%. Interestingly, higher concentrations of PEG-FA functionalization negate this effect.

**FIGURE 4:**
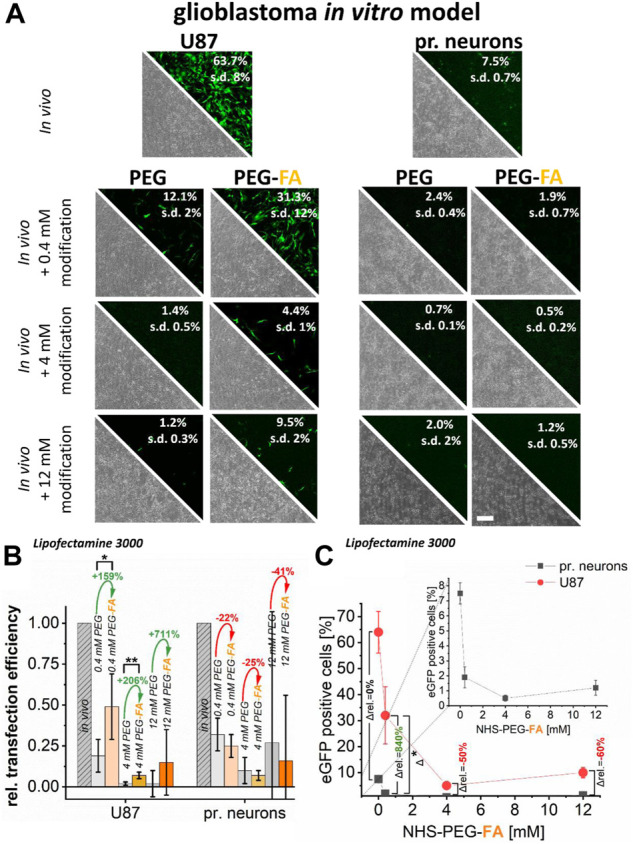
Effect of pure PEGylation and additional FA-functionalization on transfer efficiencies. In U87 glioblastoma cells and primary cortical neurons, eGFP plasmid was transferred using Lipofectamine^®^ 3000 (*in vivo* applicable protocol). In addition, this protocol was further modified with PEG and PEG-FA at equal concentrations. Analysis was performed by fluorescence microscopy and flow cytometry **(A)**. To demonstrate the supplemental effects of FA, relative transfection efficiencies are plotted in **(B)** and alterations of mean values are shown in green (positive) and red (negative). In **(C)**, progressions of transfection efficiencies by PEG-FA with improvements in favor of cancer cells shown in green and deteriorations in red are shown. Scale bar 200 μm. *n* = at least three independent experiments.

For Lipofectamine^®^ 3000 lipoplexes, pure PEGylation massively impaired transfer efficiency for U87 glioblastoma cells by approximately 80% and for primary neurons by almost 70% already at PEG concentrations of 0.4 mM. Higher concentrations resulted in an almost complete inhibition of DNA transfer (below 10%). In contrast PEG-FA functionalization led to significantly higher transfer efficiencies in cancer cells with up to 31% eGFP positive cells (0.4 mM PEG-FA; Figure 4A). Compared to pure PEGylation, all PEG-FA modifications showed significant and more efficient transfer efficiencies in U87 glioblastoma cells with significant differences for 0.4 and 4 mM. Relative improvements of transfection efficiencies to pure PEGylation of 150%–700% could be achieved (Figure 4B).

Transfer of DNA into primary neurons showed even more significant reductions in transfer efficiencies. Here, a maximum efficiency of 2% was achieved by all modifications, whereby no significant improvement of the transfer could be detected by additional FA-functionalization (Figure 4A). In fact, there were even opposite effects, where the additional FA-modification showed reduced relative efficiencies of −20% to −40% compared to pure PEGylation (Figure 4B).

Modification of the Lipofectamine^®^ 3000 complexes with 0.4 mM PEG-FA significantly improvement the transfection efficiencies in favor of U87 cancer cells compared to healthy primary neurons of more than 800% compared to unmodified complexes. However, the effects of 0.4 mM PEG-FA modification still allowed efficient transfections into the FR-overexpressing cancer cells, whereas in primary neurons they only resulted in transfection efficiencies in the range of maximum 1%. The effect of favored and selective transfection for cancer cells is negated again with increasing PEG-FA concentration (4–12 mM), so that a more selective DNA transfer into cancer cells is reduced.

### Reducing cell surface binding capacity of FA-conjugated lipoplexes—Analysis of folate receptor dependent DNA-transfer

**FIGURE 5:**
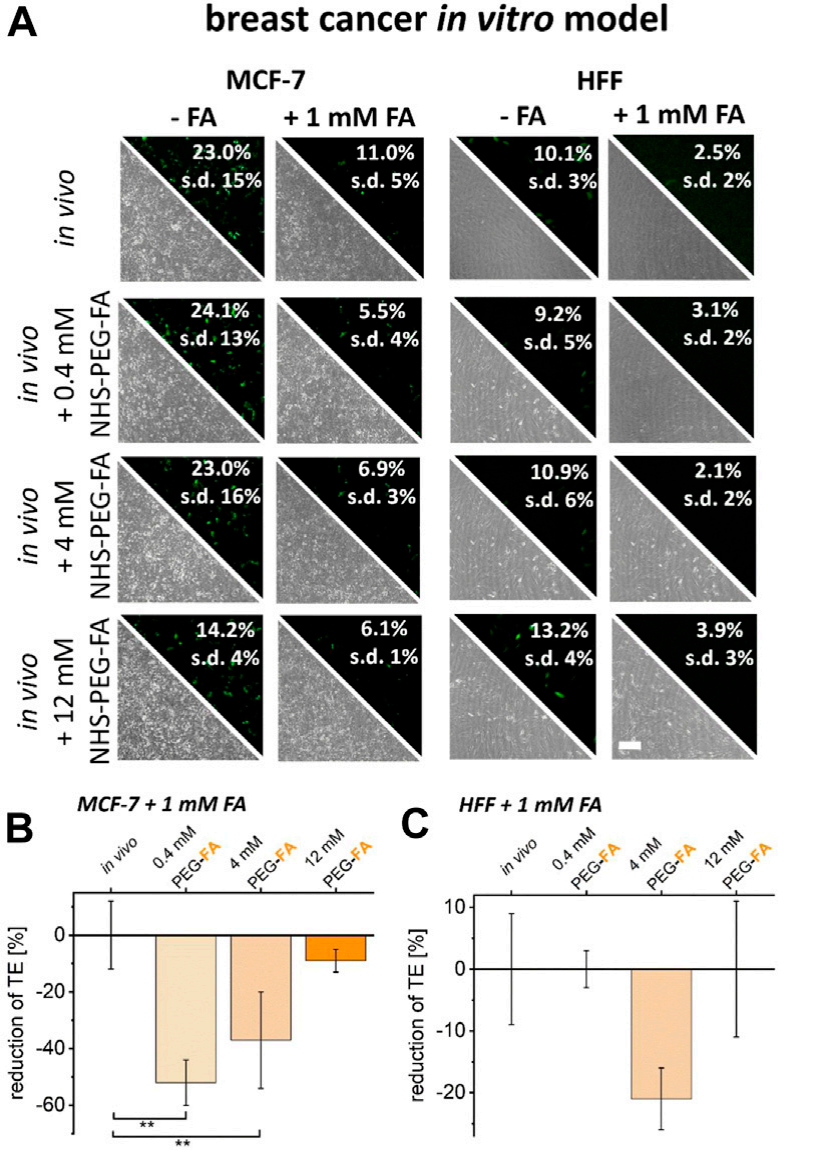
Reduction of FA binding capacity and the impact on transfer efficiencies of FA functionalized lipoplexes. To reduce the uptake capacity of FA- functionalized lipoplexes, cancer cells (MCF-7) and healthy HFF cells were pre-incubated with 1 mM FA. The resulting transfer efficiencies were analyzed by fluorescence microscopy and flow cytometry **(A)**. The reduction in transfection efficiencies of FA-functionalized lipoplexes was characterized comparatively between unmodified *in vivo* protocols and PEG-FA functionalized lipoplexes (0.4–12 mM) for cancer cells **(B)** and healthy cells **(C)**. Effects were normalized to the influence of free FA incubation on the *in vivo* protocol. Scale bar 200 μm. *n* = at least three independent experiments.

To verify folate receptor-dependent transfer of PEG-FA functionalized lipoplexes into cancer cells, surface binding capacity of FA-modified lipoplexes was reduced by addition of free FA to culture medium prior to transfection. As a result, FA supplementation already reduced transfer efficiencies of unmodified lipoplexes (Figure 5A, *in vivo* protocol) for healthy as well as cancer cells.

For PEG-FA functionalized lipoplexes, no additional reduction in transfer efficiency could be detected for healthy HFF cells upon FA supplementation (Figure 5A, B). In contrast, for MCF-7 cancer cells a significantly stronger reduction of transfer efficiencies was observed for 0.4 mM and 4 mM of PEG-FA modifications after reduction of the surface binding capacity of FA conjugated lipoplexes. Here, the specific uptake of PEG-FA functionalized lipoplexes into cancer cells was additionally reduced by 52% (0.4 mM PEG-FA) to 37% (4 mM PEG-FA) (Figure 5B).

**FIGURE 6:**
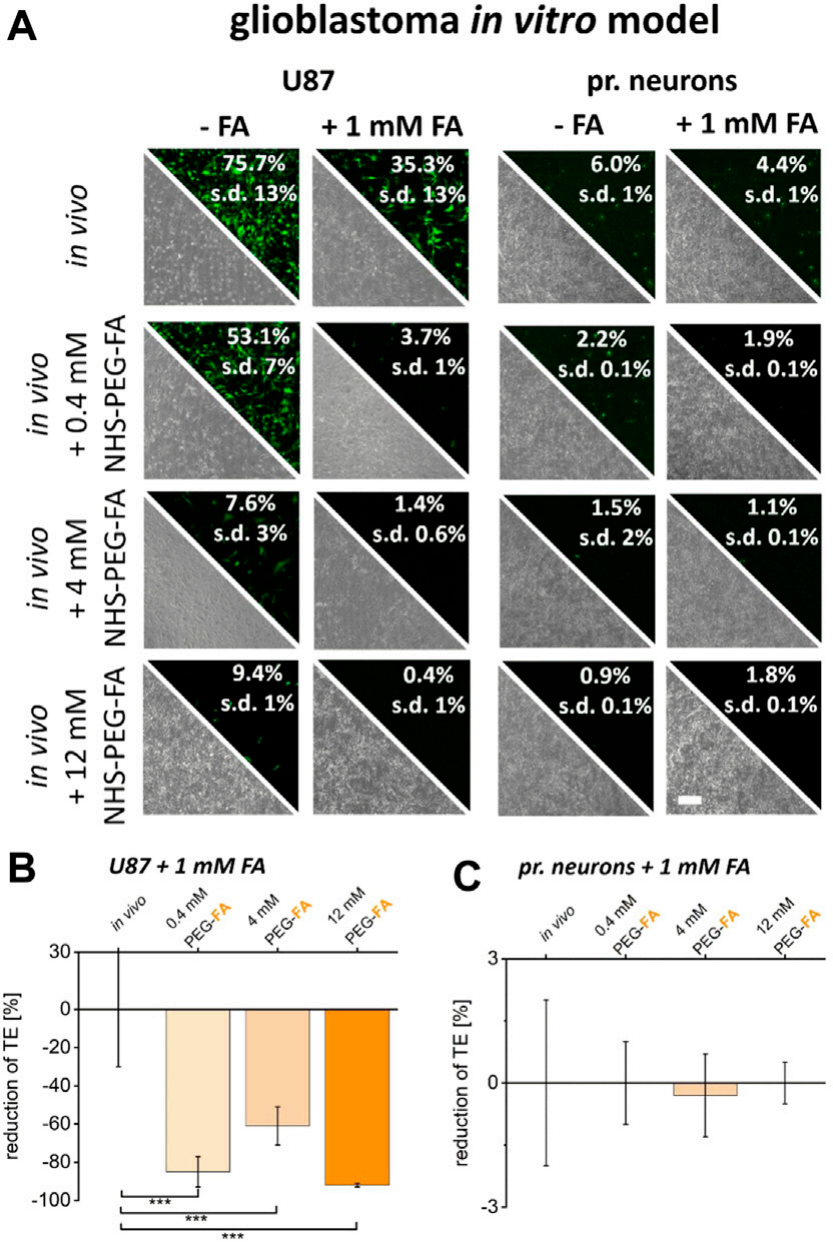
Reduction of FA binding capacity and the impact on transfer efficiencies of FA functionalized lipoplexes. To reduce the uptake capacity of PEG-FA functionalized lipoplexes, cancer cells (U87) and healthy primary cortical neurons were pre-incubated with 1 mM FA. The resulting transfer efficiencies were analyzed by fluorescence microscopy and flow cytometry **(A)**. The reduction in transfection efficiencies of FA-functionalized lipoplexes was characterized comparatively between unmodified *in vivo* protocols and FA functionalized lipoplexes (0.4–12 mM) for cancer cells **(B)** and healthy cells **(C)** normalized to the influence of FA incubation on the *in vivo* protocol. Scale bar 200 μm. *n* = at least three independent experiments.

Identical experiments were also performed on U87 cancer cells and healthy primary neurons. As before, also here free FA supplementation already reduced transfection efficiencies. Furthermore, upon use of PEG-FA functionalized lipoplexes, FA supplementation did not lead to pronounced reductions in transfer efficiencies into primary neurons independent on PEG-FA concentration (Figure 6A, C). In contrast, for U87 cells FA supplementation led to a strong additional reduction in transfer efficiencies of up to 85% (Figure 6B). These data therefore clearly confirm the folate receptor dependent and cancer cell targeted transfer of PEG-FA functionalized lipoplexes.

### FA-functionalized lipoplexes for improved transfer of DNA into FolR overexpressing cancer cells

**FIGURE 7:**
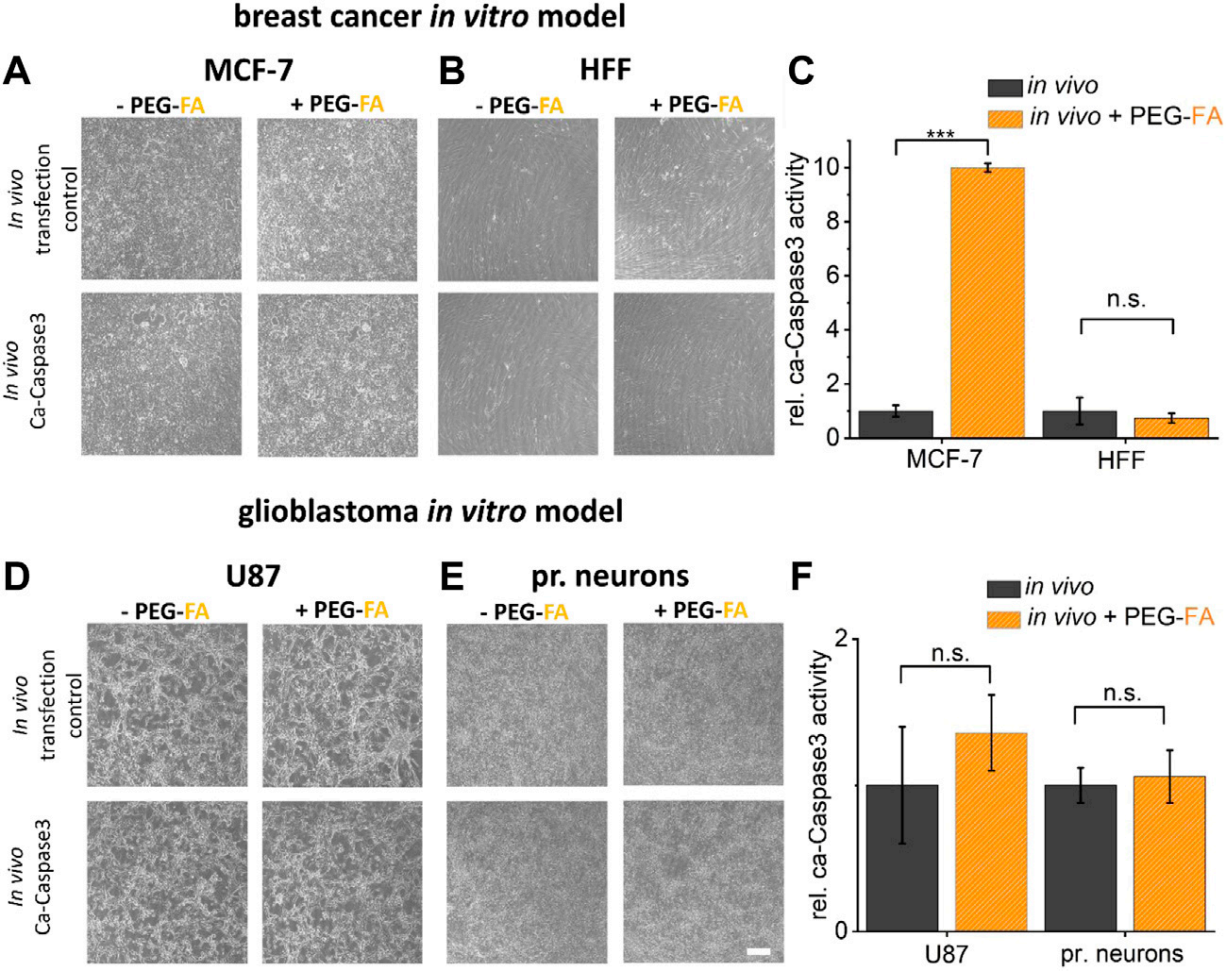
Therapeutic potential characterization by *in vitro* assays. To investigate the therapeutic potential of the functionalization developed here, a ca-Caspase3 was transferred using FA-functionalized lipoplexes (**(A), (B)** fusion/**(D), (E)** lipofection). Quantification of ca-Caspase3 activity in target and non-target cells is shown in **(C)** and **(F)**. Scale bar 200 μm. *n* = at least three independent experiments.

Finally, to analyze the PEG-FA conjugated lipoplexes optimized and characterized here in terms of their functionality and selectivity, the transfer of DNA was analyzed in both *in vitro* cancer models. PEG-FA conjugated variants of Lipofectamine^®^ 3000 and Fuse-It-DNA were used in comparison to non-modified reagents to enhance green labeling of cancer cells and to preferentially induce apoptosis of cancer cells with ca-Caspase3 constructs (Figure 7A–D).

As shown in Figure 7A–D, the influence of pure transfection on cell viability was first examined using a transfection control (analogous to previous experiments eGFP-plasmid transfection). Here, comparable transfer efficiency ratios with analogous reductions in eGFP expression of PEG-FA functionalized complexes compared to unmodified lipoplexes were shown. By using therapeutically effective ca-Caspase3 plasmids, an obvious change in cell morphology and confluence could be determined in MCF cells. In addition, a significantly improved Caspase activity was shown by using functionalized complexes. Healthy HFF cells showed no significant changes, with constant low activity of ca-Caspase3 (Figure 7C). In contrast, in the glioblastoma model, transfer of a ca-Caspase3 either did not show changes in cell morphology and density nor significant enhanced apoptosis in cancer cells was detected (Figure 7F).

### Long term stability characterization of PEG-FA functionalized lipoplexes

**FIGURE 8:**
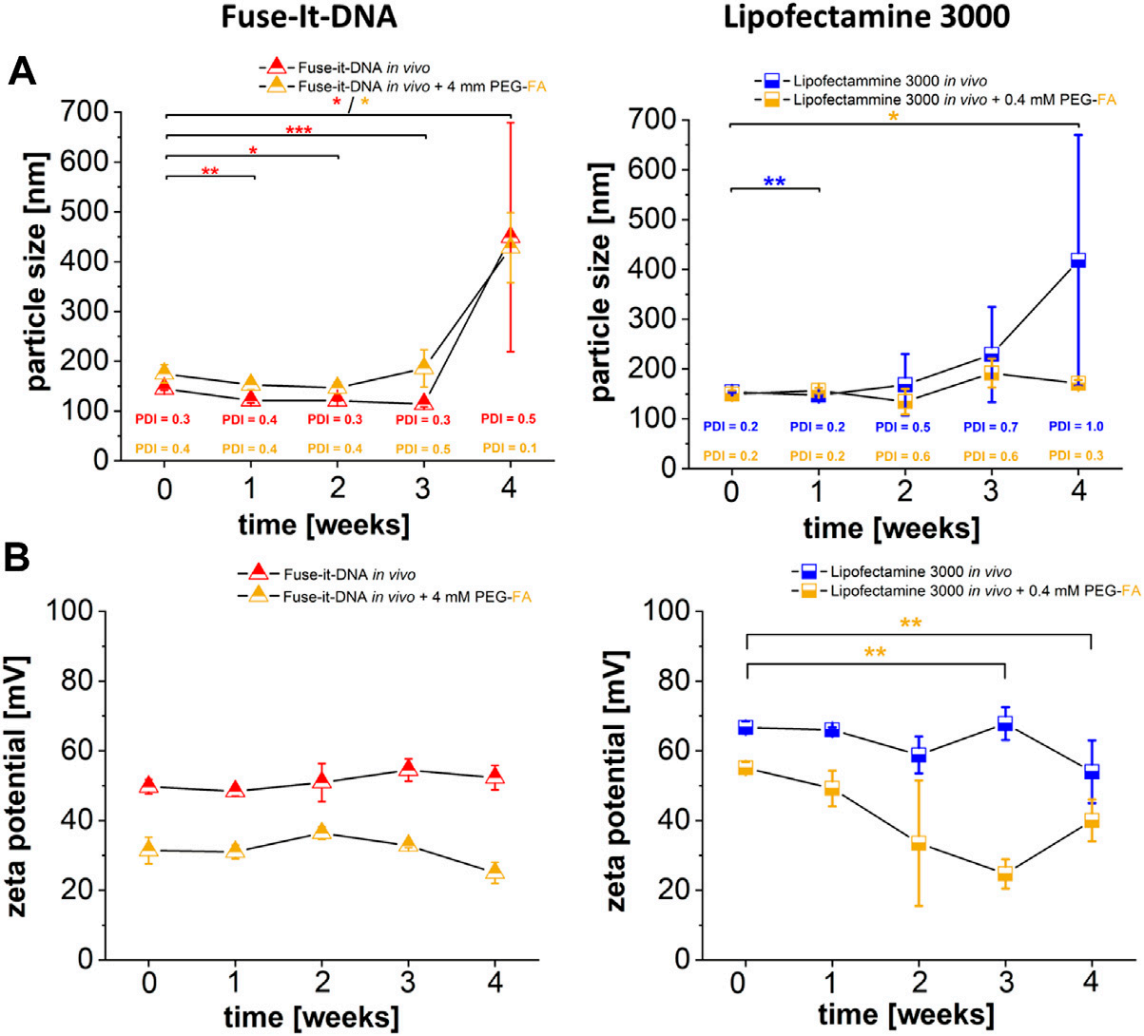
Characterization of PEG-FA functionalized lipoplexes. Physicochemical characterization of FA functionalized complexes compared with unmodified lipoplex-variants are shown in **(A)** (particle size) and **(B)** (zeta potential). *n* = at least three independent experiments.

To assess the stability of NHS chemistry-induced coupling of PEG-FA to the different liposomal transfection reagents, particle size and corresponding zeta potentials were measured over 4 weeks for both systems. There was no significant change in particle sizes for the two delivery systems after PEG-FA functionalization for 3 weeks. Only after 4 weeks, slight changes in complex size after storage at 4°C could be detected (Figure 8A). The zeta potentials showed reduced values for both functionalized variants of the lipoplexes compared to non-functionalized complexes of the same reagents. The PEG-FA functionalized variants showed no significant changes in zeta potentials in the Fuse-It-DNA reagent, while Lipofectamine^®^ 3000 PEG-FA complexes also had significantly changed values after 3 weeks (Figure 8B).

## Discussion

Functionalization of lipoplexes for targeted transfer of nucleic acid into diseased cells may represent a crucial step for the development of effective therapeutics. The use of nucleic acids for therapeutic purposes is becoming increasingly important; from immunizations (Liu et al., 2020; Walsh et al., 2020) to local and systemic cancer gene therapies (Dirisala et al., 2014; Sahin et al., 2014; Goklany et al., 2019; Sousa et al., 2019; Tang et al., 2019; Tockary et al., 2019; Hager et al., 2020; Neves et al., 2020), a wide variety of approaches are being pursued on basis of these active compounds. The reproducible preparation and use of highly concentrated lipoplexes also poses a critical challenge preclinically (Hoffmann et al., 2022) and in clinical models (Geetha-Loganathan et al., 2011), which we were able to successfully establish here with the *in vivo* protocols for Fuse-It-DNA and Lipofectamine^®^ 3000. For the latter, we additionally applied such system successfully for intratumoral glioblastoma treatments in mice (Hoffmann et al., 2022). Functionalization of liposomal formulations with FA for targeting FR overexpressing cancer cells is a well-known approach. Lee et al. already showed that improved uptake of liposomal formulations in cancer cells is possible *via* FR-associated transfer (Lee and Low, 1995). The expression levels of FR proteins shown in our data are well comparable to those already observed for mRNA quantifications with up to 18-fold increased values compared to healthy cells (Cheung et al., 2016). Thus, this approach is a promising and widely applicable technique, as this functionalization of lipoplexes could also be easily extended to other diseases by analogous high expression patterns in almost all cancer cells (Kelemen, 2006; Liang et al., 2019).

Stabilization of lipoplexes *via* PEGylation has been used successfully and is widely applied (Ogris et al., 1999; Chan et al., 2012; Shibata et al., 2015; Nosova et al., 2019). It is well-known that this PEGylation leads to a massive reduction in transfer efficiency, the so-called PEG dilemma (Mishra et al., 2004; Hatakeyama et al., 2013; Ge et al., 2014; Fang et al., 2017). In the approach shown here, this serves to reduce the nucleic acid transfer efficiency in healthy cells, while the additional FA functionalization could enable a renewed improvement of transfer efficiencies in FR overexpressing cancer cells. The improvements of transfer efficiency by PEGylation in HFF cells, which were not to be expected to this extent (see 4 mM PEG - fusion lipoplexes) could be related to specific cell-lipoplex interactions, which have also been described for other complexes (Masotti et al., 2009; Resina et al., 2009).

The various impact of PEGylation with NHS-coupled PEG and PEG-FA groups on endocytic and fusogenic lipoplexes could be attributed to their different transfer mechanisms. While classical lipoplexes are taken up *via* endocytosis, fusogenic lipoplexes show a different transfer path in which the liposome actively fuses with the cell membrane and transfers the nucleic acid into the cytoplasm (Hoffmann et al., 2019; Hoffmann et al., 2020). Vanic et al. previously demonstrated that PEGylated lipids can be used for the preparation of fusogenic lipoplexes. Although these lead to a reduction in fusion efficiency, fusion processes can still be observed (Vanic et al., 2012). This could explain the reduced influence on the Fuse-It-DNA complexes since an active fusion process may still be possible at concentrations of PEG and PEG-FA used here. For Lipofectamine^®^ 3000, decreasing transfer efficiencies are already observed at lower concentrations. Since these lipoplexes are exclusively taken up *via* endocytosis, the influence of FA-FR interactions on transfer efficiencies seem to be more evident. FA-FR interactions also induce endocytic uptake mechanisms (Zhao et al., 2014; Allard-Vannier et al., 2017; Kumar et al., 2019), which may replace the main transfer pathway of these lipoplexes. As a result, this system is very efficient in reducing side effects and almost preventing nucleic acid transfer in healthy cells, since no processes, as for fusion, can lower the promoted effects.

The effect of PEGylation on the stability of the complexes *in vivo* could not be demonstrated in this study. However, an improvement in the stability of the lipoplexes generated here seems likely, as post-insertion methods have been shown to improve the half-life of lipoplexes in serum by up to 30-fold (Anselmo et al., 2015).

The reduction of binding affinities of FA-functionalized lipoplexes to cellular FR was performed by saturation of the culture medium with 1 mM FA, as already performed by Reddy et al. (Reddy et al., 2002). The significant reduction of the uptake of FA functionalized lipoplexes exclusively in cancer cells strongly argues for the selective uptake of FA functionalized lipoplexes established here. The reduction of non-FA functionalized lipoplex uptake has been shown analogous (Reddy et al., 2002) and can be induced by several processes. On the one hand, the strong FA binding to cellular FR could prevent the uptake of lipoplexes by masking cell membranes and reduce the interaction of lipoplexes*.* Moreover, a prioritization of FA-FR induced endocytosis in comparison to endocytic uptake mechanisms of lipoplexes is possible. On the other hand, FA molecules and FA-functionalized lipoplexes could electrostatically interact in the culture medium, resulting in reduced binding affinities of the lipoplexes with cell membranes.

The transfer of the system shown here to an *in vitro* preliminary study on the treatment of cancer cells with a ca-Caspase 3 showed for the fusion system a comparative efficiency with increased selectivity compared to the previous data and thus potentially therapeutic use for targeted apoptosis induction in cancer cells with significantly reduced effects on healthy cells of treated tissues. These results could not be shown analogously for the glioblastoma model and Lipofectamine^®^ 3000. Here, cell-specific intracellular processes can also reduce the Caspase activity, since on the other hand the selectivity of the eGFP transfections could be identically improved (Stegh et al., 2008; Shen et al., 2016). In order to ensure highest possible pDNA transfer for potential therapeutic application, both transfection reagents were investigated in resulting transfer efficiencies prepared according to the manufacturer’s protocol for all cell types used here. The cell-specific effects shown here with respect to transfection efficiency and biocompatibility of the different reagents may be due to product-specific composition and resulting interaction with differently composed cell membranes. These effects could also be demonstrated in previous studies (Hoffmann et al., 2019). In general, the use of commercially available reagents can be profitable for such applications. There is already a large amount of data and results for both systems that can be used to further optimize these mixtures (Csiszar et al., 2010; Kleusch et al., 2012; Cardarelli et al., 2016; Gonzalez Villarreal et al., 2018; Kolasinac et al., 2018; Wang et al., 2018; Hoffmann et al., 2019; Kolasinac et al., 2019; Hoffmann et al., 2020). In addition, these reagents are manufactured under highest quality standards, which ensures reproducible and consistent preparation. In addition, the functional conjugation of post-insertion and commercially available reagents demonstrates an easy and fast adaptable method to design specific nucleic acid delivery systems without redesigning entire liposome formulations. Even if the exact compositions are not known, targeted modification and functionalization can be carried out with these reagents, as shown here.

The functionalization of the reagents used here also exhibits very comparable alterations in their physicochemical characteristics as described before. As already shown in previous studies, lipoplexes remain quite small in their size distributions after post-insertion method and do not show significant changes in lipoplex diameters (Nosova et al., 2019). Also analogous to the data shown here, it was previously shown, that lipoplexes after PEGylation exhibit a reduction of zeta potentials with increasing PEG concentration (Chen et al., 2013; Hashimoto et al., 2014; Hattori et al., 2020). This alterations of zeta potentials also have massive effects on transfer efficiencies of endocytotic lipoplexes (Son et al., 2000). Therefore, the reducing transfer efficiencies with increasing PEG and PEG FA concentrations for Lipofectamine^®^ 3000 are closely related to reduced zeta potentials. Although fusion lipoplexes are very dependent on their zeta potential for efficient transfer of nucleic acids (Hoffmann et al., 2020), these lipoplexes show minor influences on PEGylation and quite stable transfer efficiencies despite significant reduced zeta potentials. This could suggest that a change in the transfer mechanism or an even more extensive change in the Fuse-It-DNA based lipoplex structures is involved (Kolasinac et al., 2019). However, this could not be conclusively considered in this publication.

In addition, the *in situ* stability and characterization of the complexes over a period of 4 weeks shown here, indicate that the functionalization of both lipoplexes are very stable and efficient. Also, Resnier et al. (Resnier et al., 2019) and Cheng Wei Chen et al. (Chen et al., 2013) assume that a 3 weeks stability of lipoplexes with PEG coupled components is supportive for a stable lipoplex production. Thus, the method developed here for Lipofectamine^®^ 3000 and Fuse-It-DNA not only demonstrates practical protocols for *in vivo* application of high-concentration DNA lipoplexes but also that the proposed modification method could provide an efficient functionalization tool for both systems. This aspect in combination with first applications *in vivo* could provide crucial insights into the improved selectivity and serum stability. Since both reagents have already shown significant improvements for selectivity in the cell culture-models shown here, first *in vivo* applications for targeted nucleic acid therapy approaches are the next mandatory step.

## Data Availability

The raw data supporting the conclusions of this article will be made available by the authors, without undue reservation.
